# Effects of thyroid hormone on HSV-1 gene regulation: implications in the control of viral latency and reactivation

**DOI:** 10.1186/2045-3701-1-24

**Published:** 2011-07-14

**Authors:** Shao-Chung Hsia, Gautam R Bedadala, Matthew D Balish

**Affiliations:** 1 Department of Pharmaceutical Sciences, University of Maryland, Eastern Shore School of Pharmacy, Princess Anne, Maryland 21853, USA

## Abstract

Thyroid hormone (TH) is involved in many biological functions such as animal development, cell differentiation, etc. Variation and/or disruption of plasma TH level often led to abnormalities and physiological disorders. TH exerts the effects through its nuclear receptors (TR). Literature showed that procedures resulted in TH alteration also linked to reactivation of several viruses including Herpes Simplex Virus Type -1 (HSV-1). Bioinformatic analyses revealed a number of putative TH responsive elements (TRE) located in the critical regulatory regions of HSV-1 genes such as thymidine kinase (TK), latency associated transcript (LAT), etc. Studies using neuronal cell lines have provided evidences demonstrating that liganded TR regulated viral gene expression via chromatin modification and controlled viral replication. The removal of TH reversed the inhibition and induced the viral replication previously blocked by TH. These results suggest that TH may have implication to participate in the control of reactivation during HSV-1 latency.

## 1. Thyroid Hormone Overview

Thyroid hormone (TH or T_3_) contributes to numerous crucial physiological processes ranging from animal development, proliferation, differentiation, apoptosis, etc [1]. Aberration due to the lack of TH led to disorders such as the goiter (thyroid gland enlargement) and cretinism (a type of severe mental retardation) [2]. TH deficiency may result from the shortage of iodine (essential for TH biosynthesis), thyroidectomy, diseases, or inherited defects, etc. In humans, developmental defects because of TH deficiency can be treated by TH replacement at appropriate time frame [3].

## 2. Transcriptional regulation by TR

TH is vital for the normal functions of many organs and exert its ability through the nuclear receptor [1]. Two TR genes (TRα and TRß) were identified in vertebrates and both exhibit strong binding to their ligand TH [4-7]. They are members of nuclear hormone receptor super-family [8-10]. Although recent discovery suggested non-genomic action, TRs primarily produce their activity by binding to TH response element (TRE) and regulating transcription in nucleus through the status of TH [11]. TRE is a short DNA sequence located within the promoter of a TH-response gene. The most common TRE sequence is a pair of direct repeats separated by four nucleotides (DR4), indicating that the correspondent receptors bound as a dimer [12]. This TRE is also described as positive TRE since transcription activated by TH requires the interaction of liganded TRs to the DR4-TRE within the promoter of TH-response genes, most likely as heterodimers with RXRs (9-cis retinoic acid X receptors). TR/RXR heterodimers display constitutive interaction to DR4-TRE in chromatin context regardless the status of TH [13]. In the absence of TH, they inhibit the gene expression and the inhibition can be reversed when TH is available for binding to the receptors [13]. TR controls gene expression by recruiting cofactors to the vicinity of promoters. The corepressors are preferentially recruited by unliganded TR while the coactivators are enriched at the promoter via liganded TR [13]. The characterization indicated that corepressors such as SMRT, N-CoR, etc establish complexes with histone deacetylases (HDACs) to facilitate hypoacetylation. This process eliminates those acetyl groups on histone tail, increases the positive charge of nucleosomes, and enhances the interaction between the histones and DNA backbone. As a result, it promotes the chromatin binding and condenses DNA structure, therefore preventing transcription [14]. On the contrary, many coactivators (e.g. SRC-1, CBP/p300) are histone acetyltransferases (HATs) and induce transcription by reversing the previous process [14].

## 3. Herpes Simplex viruses (HSV) and diseases

HSV is one of the most common causes of infectious disease in humans [15]. This virus contains two distinct types, HSV-1 and HSV-2. Their genomes demonstrate approximately 50% homology. Diseases caused by HSV-1 infection appear frequently [16]. It happens to children about 3 to 5 years old and lasts 5 to 12 days [17]. After the initial infection, the virus may establish latency in the trigeminal ganglia. Reactivation usually occurs over the anterior mucosa, lips or perioral area of face called cold sores or fever blisters [18]. About 10% of viral encephalitis resulted from herpes virus [19]. The factors leading to HSV encephalitis are unknown although a study suggested that latent virus reactivation in the trigeminal ganglia and prolonged lytic infection in the temporal-parietal area of the brain may play a role [20]. Other major clinical syndromes include cornea infection (herpes keratitis) [21], infection of finger and nail (herpetic whitlow) [22], herpes dermatitis [23], and genital HSV infection [24].

## 4. The genome of HSV-1 and gene expression

### a. Genome

HSV-1 has a linear double-stranded DNA genome. The genome contains approximately 80-85 genes and is about 152 kbp long. The G+C content of HSV-1 genome is approximately 68% [25]. It includes two components, designated as L (long) and S (short). Both L and S contain unique sequences, designated as U_L _and U_S_, and each of them is bracketed by inverted repeats. The L and S of HSV-1 can invert with respect to one another, yielding four linear isomers. The isomers are defined as P (prototype), I_L _(inversion of the L component), I_S _(inversion of the S component), I_SL _(inversion of S and L) [26].

### b. Characterization of gene expression

Gene expression of HSV-1 is tightly regulated in a cascade fashion. The three temporal classes of genes are designated immediate-early (α), early (β) and late (γ) genes [27]. There are five α genes and their products are often named Infected Cell Protein (ICP), designated ICP0, ICP4, ICP22, ICP27, and ICP47. All of these proteins have regulatory functions except ICP47, which inhibits major histocompatibility complex class I antigen presentation [28,29]. The α genes are defined by the presence of the sequence 5'-TAATGARATT-3' (the cis element for induction of α genes by VP16) upstream of the cap site [30]. VP16 interacts with cellular factors, including the protein Oct-1, a homeobox protein, to activate viral α gene transcription in trans [31]. The expression of β genes requires the expression of α genes [32].

## 5. HSV-1 latency, reactivation, and its regulation by TH

### a. Latency and reactivation

HSV-1 establishes latent infections in peripheral nerve ganglia following primary infection in the cells of mucosal membranes or skin [33]. Latent infection is maintained lifelong in the human host. The virus may reactivate from time to time and infectious virus enters peripheral tissues by axonal transport causing recurrent disease or subclinical virus shedding [34]. Latent virus may be reactivated after local or systemic stimuli such as injury to tissues innervated by neurons harboring latent virus, or by emotional or physical stress [35,36]. During latency, the viral transcription is restricted to a region within the long terminal and internal repeats and the transcripts are designated as "LATs" (Latency Associated Transcripts) [37]. The molecular functions of LAT during latency and reactivation are elusive and the specific roles in gene regulation are not exactly understood. Some suggested that LATs produced anti-apoptotic effects [38]. In neuronal cells, LAT was shown to reduce viral gene expression and replication during productive infection [39]. *In vivo*, LAT mutant virus enhanced gene expression in sensory neurons during lytic and latent infection [40]. Additional report showed that LAT augments transcriptions of several lytic genes during the latent stage in rabbits [41]. Since they are the only major transcripts produced in significant amounts during latency, the LATs were suggested to play a role in establishing, maintaining, or reactivating latency [42].

During reactivation, LAT gene decreased and was shown to be associated with repressive histones [43]. Transcripts of ICP0, on the other hand, accumulated and the histones around its promoter became acetylated [43]. These results suggested the roles of LAT and ICP0 during the initial stage of reactivation. As for the viral gene expression profile in neurons during reactivation, it was suggested to follow the paradigm of α to β to γ cascade and viral DNA replication occurred after α synthesis, similar to the lytic cycle. However, other reports challenge this view by suggesting different sequence. For example, studies showed that TK-minus mutant exhibited greatly reduced α and β expression during reactivation [44,45]. These observations were further supported by the finding that TK, a β gene, was detected before α gene expression using explants reactivation model [46]. In addition, viral replication is required for efficient α and β expression in neuron during reactivation [47]. Together, these studies emphasized the importance of TK and viral DNA synthesis during reactivation. Since TK is required to provide dNTP for viral replication in resting cells such as neurons, it is likely that TK may play a critical role to stimulate viral DNA synthesis and α gene expression to promote reactivation.

### b. Role of TH on HSV-1 reactivation

The impacts of TH on virus-mediated pathophysiology was discussed but not extensively studied. Low serum thyroxine and other hormone imbalance due to hypothalamic-hypopituitarism were associated with viral meningo-encephalitis and related complication [48]. Effects of TH on AIDS (Acquired Immuno-Deficiency Syndrome) and ARC (AIDS-Related Complex) were investigated and the results suggest that TH may affect disease development and progression [49]. At present, the molecular basis of the HSV-1 latency/reactivation is not extensively understood. In particular, it is not known why virtually the entire HSV genome is transcriptionally silent, with the exception of the LAT region. It is not entirely clear how the latent virus initiates gene expression/replication upon reactivation. Recent studies suggested that TH and TRs played roles on HSV gene silencing/activation and DNA replication during latency/reactivation [50,51]. There are reasons to hypothesize that the status of TH and its interaction with TRs modulate chromatin and exert functions in HSV-1 latency and reactivation. 1. TR is present in ganglia neurons [52,53]. 2. TH can affect different biological processes involved in the survival, differentiation, maturation of neurons [54]. 3. TH and nerve growth factor enhanced neurite outgrowth, and regulate the expression of dynein, a protein that is involved in axonal transport (important for virus movement), in ganlia neurons [55]. There is no direct, controlled clinical study regarding the effect of TH on HSV-1 reactivation although alteration of corticosteroid has been linked to HSV-1 reactivation [56,57]. A case study showed that a patient with myxedema coma under corticosteroid treatment developed herpes simplex encephalitis with extremely low thyroxine level less than 5.2 nmol/L (normal range 12-30 nmol/L) [58]. In addition, literature indicated that many factors, such as stress, febrile diseases, trauma, surgery, radiotherapy, etc, triggering HSV-1 reactivation also altered thyroid hormone level (Table 1).

**Table 1:** Comparison between triggering factors of HSV-1 reactivation and thyroid hormone alteration

	HSV-1 Reactivation	Change in Thyroid hormone levels
Stress	[59-63]	[64-68]

Fever	[69-72]	[73-76]

Local injury to face lips or eyes	[77-79]	[80]

Trauma	[81-83]	[84-86]

Surgery	[81,82,87-91]	[80,92,93]

Radiotherapy	[94,95]	[96-98]

This table is intended to provide connection between variation of TH level and HSV-1 reactivation. For example, whole body hyperthermia was reported to reduce the level of serum TH by 50%, probably due to the suppression of thyroid stimulating hormone release, monodeiodination alteration of T_4 _from TH to reverse T_3_, and enhanced TH clearance [76]. Importantly, hyperthermia is regularly used by laboratories to trigger HSV-1 reactivation in the mouse latency model [72]. In addition, brain injury and trauma were reported to reduce TH levels [86] and also trigger viral reactivation (see Table 1). Therefore, TH is likely to participate in the regulation and maintenance of viral latency and reactivation. TH acts on almost every cell in the body including neurons. It is likely that TH contribute, at least in part, to the regulation of HSV-1 latency/reactivation.

### c. Characterization of HSV-1 TKTRE

**Figure 1:**
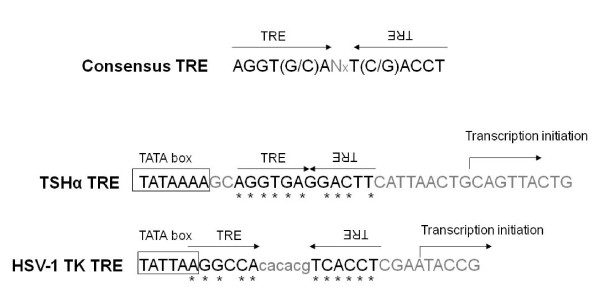
**Characterization of HSV-1 TK TREs**. Comparison of HSV-1 TK TREs to other palindrome TREs. These TREs were organized as inverted repeats with different numbers of nucleotides spacing in between them (see Consensus TREs). They are located after the TATA box and in front of the transcription initiation site (see TSHα TREs and TK TREs).

Early report revealed a pair of TRE located in the HSV-1 TK promoter and produced positive regulation [99]. Additional analyses indicated that these TK TREs are positioned between TATA box and the transcription initiation site arranged as "palindromes" with six nucleotides spacing each other (Figure 1). TREs in this format were also found in the promoters of TSHα and TSHβ, both exhibit negative regulations by TH in hypothalamic-pituitary-thyroid (HPT) axis [100]. It has been suggested that this TK TREs exhibited negative regulation by TH and TR in neuronal environment [51,99].

#### i. TR negatively regulated HSV-1 TK transcription in neuronal cells

Analyses using mouse neuroblastoma cell lines N2a and N2aTRβ showed that HSV-1 TK promoter activity was repressed by liganded TR and activated in the presence of TR without TH using transient transfection assays [51]. It appears that TR exerted negative regulation on HSV-1 TK promoter in a neuronal cell line since the regulation was not observed in other cell lines such as 293HEK and Vero (data not shown). The binding of TR to negative TREs and the outcome of histone modification in neuronal cells were not well characterized. Chromatin immunoprecipitation (ChIP) assays showed that liganded TR exhibited strong interaction to the TK promoter via TREs. The binding was significantly reduced in the absence of TH [51]. Hypoacetylation was observation at the TK promoter by TH and TR using anti-acetyl H4 Ab [51]. These results indicated that liganded TR was recruited to the TK promoter and reduced the acetylation of histone tails at the TRE region.

#### ii. TK was repressed by TH and TR during viral infection

Based on the results of transfection assays, infections of cells with viruses were performed to investigate the TR/TH mediated regulation in neuronal cells. RT-PCR assays showed that TK transcription was inhibited by liganded TR at high moi while the viral protein synthesis was inhibited (unpublished data). It is noted that the TK promoter activity was efficiently repressed by TR/TH at low moi [51]. These results demonstrated that liganded TR repressed TK promoter activity in neuronal cells through chromatin modification via interaction with TK TREs.

#### iii. Liganded TR mediated TK inhibition can be reversed by TH removal

It was hypothesized that increasing TK expression may enhance HSV-1 replication/gene expression thus promoting viral DNA replication and α expression during reactivation [47]. Results from N2aTRβ cell culture model indicated that the removal of TH de-repressed the TK inhibition [51]. In addition, the same condition of TH washout can reactivate the expression of ICP0 [50], another important HSV-1 α gene for reactivation. Together these results further support the hypothesis that TH may have implication in the HSV-1 reactivation from latency through the induction of TK and ICP0.

## 6. In vitro TH-mediated HSV-1 latency cell culture model

**Figure 2:**
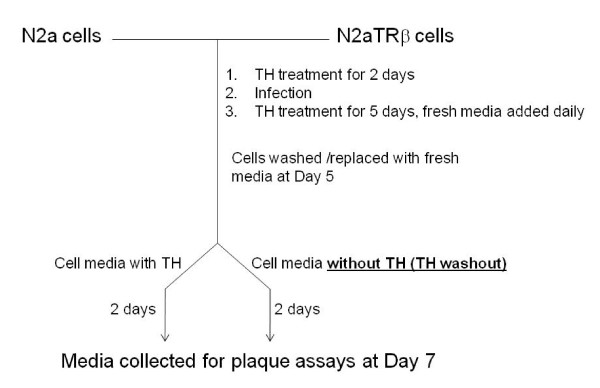
**Establishment of a TH-regulated HSV-1 latency/reactivation cell culture model**. Scheme of cell culture based HSV-1 latency/reactivation model.

A cell culture model was established to investigate the roles of TH/TR in the regulation of HSV-1 latency/reactivation [50,51] (depicted in Figure 2). This system is based on the fact that over-expression of TR isoform β triggers N2a cells to differentiate in the presence of TH [101], mimicking the state of neurons where HSV-1 established latency. The plaque assays indicated that release of infectious virus was significantly decreased in the presence of TH with TR. Furthermore, TH washout de-repressed the virus replication and release [51]. These observations demonstrated the previous findings that TH availability played roles in the regulation of HSV-1 reactivation/latency.

## 7. Conclusion and future direction

**Figure 3:**
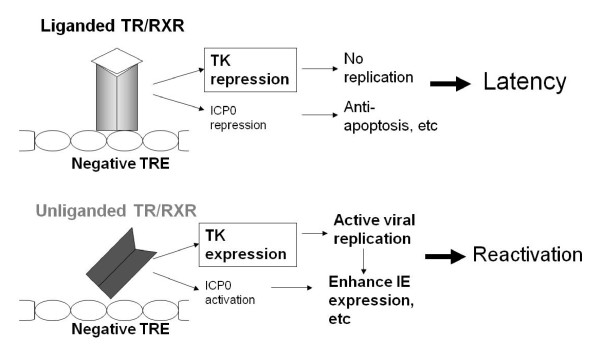
**Model of TR/TH-mediated HSV-1 latency and reactivation**. The working hypothesis is that liganded TR repressed the transcription of TK in neurons, leading to inhibition of viral replication and α expression thus promoted the condition for latency. Transient or chronic hypothyroidism reduced the TH level and the shortage of hormone decreased the repression of TK and ICP0, therefore increased the viral replication, gene expression, and release of infectious viruses. All of these led to viral reactivation.

A number of studies suggested the possibilities that hormone imbalance may cause virus reactivation including HSV-1. The mouse neuroblastoma cell line N2a and N2aTRβ provided an efficient platform to investigate the molecular functions of TR and TH in the regulation of HSV-1 latency and reactivation. Current progress suggested that TR/TH inhibits the HSV-1 key gene expression, leading to blockade of viral replication and α expression therefore favors maintenance of latency in neurons. Transient or chronic hypothyroidism decreases the TH level and the lack of hormone relieves the inhibition of TK and ICP0 (likely to be indirect effect through TK activation [51] and insulator effects from the LAT regulatory region [50]), results in viral replication, gene expression, release of infectious viruses, and viral reactivation (Figure 3). Additional studies showed that this TR/TH-mediated regulation was due to, at least in part, by histone modification [50,51]. In the future, the hypotheses should be tested in the animal models using TH and TR-selective thyromimetic agents. This will assist in explaining the roles of TH on the maintenance of viral latency, probably by blocking the α activation in latently-infected mice upon reactivation. Conversely, treatment with TH antagonists can be tested to see if they have effects on the expression of HSV-1 ICP0 and TK, viral replication, and viral reactivation. Standard established protocols such as hyperthermia will be used for reactivation and eye swabs will be taken at the appropriate times for analysis of reactivated infectious HSV-1. Trigeminal ganglia neurons from the treated infected mice will be removed for analyses of gene expression, chromatin remodeling, and measurement of HSV-1 copy number. In addition, removal of TH production by thyroidectomy can be used to confirm the regulatory effects during reactivation.

## Abbreviations

HSV: Herpes simplex virus; TH: Thyroid hormone; TR: Thyroid hormone receptor; TK: Thymidine kinase; TRE: Thyroid hormone responsive elements; LAT: Latency associate transcript; ICP0: Infected cell protein 0; ChIP: Chromatin immune-precipitation; IE: Immediate early genes

## Competing interests

The authors declare that they have no competing interests.

## Authors' contributions

SVH, GRB, and MDB composed and discussed the article. All authors read, agreed, and approved the final version of this manuscript.
